# DNA Mismatch Repair Gene Mosaicism Is Rare in People With Mismatch Repair-Deficient Cancers

**DOI:** 10.1053/j.gastro.2024.12.027

**Published:** 2025-01-07

**Authors:** ROMY WALKER, JIHOON E. JOO, KHALID MAHMOOD, PETER GEORGESON, ANGELS-CCFR-MUIR-TORRE CONSORTIUM, INGRID M. WINSHIP, DANIEL D. BUCHANAN

**Affiliations:** Colorectal Oncogenomics Group, Department of Clinical Pathology, Victorian Comprehensive Cancer Centre, The University of Melbourne, Melbourne, Victoria, Australia, and Victorian Comprehensive Cancer Centre, University of Melbourne Centre for Cancer Research Melbourne, Victoria, Australia; Colorectal Oncogenomics Group, Department of Clinical Pathology, Victorian Comprehensive Cancer Centre, The University of Melbourne, Melbourne, Victoria, Australia, and Victorian Comprehensive Cancer Centre, University of Melbourne Centre for Cancer Research Melbourne, Victoria, Australia; Colorectal Oncogenomics Group, Department of Clinical Pathology, Victorian Comprehensive Cancer Centre, The University of Melbourne, Melbourne, Victoria, Australia, and Victorian Comprehensive Cancer Centre, University of Melbourne Centre for Cancer Research, Melbourne, Victoria, Australia, and Melbourne Bioinformatics, The University of Melbourne, Melbourne, Victoria, Australia; Colorectal Oncogenomics Group, Department of Clinical Pathology, Victorian Comprehensive Cancer Centre, The University of Melbourne, Melbourne, Victoria, Australia, and Victorian Comprehensive Cancer Centre, University of Melbourne Centre for Cancer Research, Melbourne, Victoria, Australia; Genomic Medicine and Family Cancer Clinic, Royal Melbourne Hospital, Parkville, Victoria, Australia, and Department of Medicine, The University of Melbourne, Parkville, Victoria, Australia; Colorectal Oncogenomics Group, Department of Clinical Pathology, Victorian Comprehensive Cancer Centre, The University of Melbourne, Melbourne, Victoria, Australia, and Victorian Comprehensive Cancer Centre, University of Melbourne Centre for Cancer Research, Melbourne, Victoria, Australia, and Genomic Medicine and Family Cancer Clinic, Royal Melbourne Hospital, Parkville, Victoria, Australia; 1 Colorectal Oncogenomics Group, Department of Clinical Pathology, Victorian Comprehensive Cancer Centre, The University of Melbourne, Melbourne, Victoria, Australia; 2 Centre for Cancer Research, Victorian Comprehensive Cancer Centre, University of Melbourne, Melbourne, Victoria, Australia; 3 Envoi Specialist Pathologists, Brisbane, Queensland, Australia; 4 Department of Molecular and Cellular Pathology, University of Queensland, Brisbane, Queensland, Australia; 5 Melbourne Bioinformatics, The University of Melbourne, Melbourne, Victoria, Australia; 6 Genomic Medicine and Family Cancer Clinic, Royal Melbourne Hospital, Parkville, Victoria, Australia; 7 Department of Medicine, The University of Melbourne, Parkville, Victoria, Australia; 8 Colorectal Medicine and Genetics, The Royal Melbourne Hospital, Parkville, Victoria, Australia; 9 Centre for Epidemiology and Biostatistics, Melbourne School of Population and Global Health, The University of Melbourne, Parkville, Victoria, Australia; 10 Public Health Sciences Division, Fred Hutchinson Cancer Center, Seattle, Washington; 11 Research Centre for Hauora and Health, Massey University, Wellington, New Zealand; 12 Division of Gastroenterology & Hepatology, Department of Medicine, Mayo Clinic, Phoenix, Arizona; 13 Center for Individualized Medicine, Mayo Clinic, Rochester, Minnesota; 14 Comprehensive Cancer Center, Mayo Clinic, Rochester, Minnesota; 15 Sullivan Nicolaides Pathology, Brisbane, Queensland, Australia.

Lynch syndrome, the most common hereditary cancer syndrome (~1 in 280 people), is caused by germline pathogenic variants in one of the DNA mismatch repair (MMR) genes, *MLH1*, *MSH2*, *MSH6*, and *PMS2*.^1,2^ People with Lynch syndrome have an increased risk of colorectal cancer (CRC), endometrial cancer (EC), and other cancers,^3^ including sebaceous skin tumors (SST).^4^ Identifying Lynch syndrome is important for clinical management and cancer prevention, but despite advances in next-generation sequencing, the detection of all pathogenic MMR gene variants remains challenging. Postzygotic mosaicism in the MMR genes is uncommon,^5,6^ but whether MMR mosaicism is truly rare or underdiagnosed due to the absence of systematic investigations is unclear.

Our aim in this study was to identify mosaic MMR pathogenic variants in people with MMR-deficient CRCs, ECs, or SSTs. We characterized 135 participants from the: (1) Applying Novel Genomic approaches to Early-onset and suspected Lynch Syndrome colorectal and endometrial cancers (ANGELS) (n = 76), (2) Colon Cancer Family Registry (CCFR) (n = 38), and (3) Muir-Torre Syndrome (MTS) (n = 21) studies who developed MMR-deficient CRCs (n = 96), ECs (n = 18), or SSTs (n = 21) for germline and somatic MMR pathogenic variants.^7^

After Lynch syndrome or *MLH1* hypermethylation was excluded as the cause of tumor MMR-deficiency, tumor and matched germline DNA sequencing identified a double (n = 119) or a single (n = 16) somatic MMR mutation.^7^ We hypothesized that 1 of these somatic MMR mutations may be a mosaic pathogenic variant for a proportion of these 135 participants. Participants provided informed consent, and the studies were approved by the University of Melbourne Human Research Ethics Committee (HREC#1750748, HREC#1954921, and HREC#1648355).

The study design is presented in Figure 1A. The CRC, EC, and SST tumor and matched blood-derived DNA for all 135 participants were sequenced on a 297-gene capture (capture A) at ~400× and ~100× coverage, respectively, as previously described.^7^ To detect low-level mosaicism, the 135 blood-derived DNA samples underwent further deep sequencing, with a median on-target coverage of 5236× (interquartile range, 3558×–7189×). For 26 of 135 cases, DNA from the normal colon or normal endometrial epithelium from the surgical resection margin specimen (“normal nonadjacent”) or normal tissue adjacent to the cancer (“normal adjacent”), or both, was tested using a custom-designed 5-gene capture (capture B: *MLH1*, *MSH2*, *MSH6*, *PMS2*, and *APC*) and sequenced to a median on-target coverage of 4056× (interquartile range, 2197×–5974×).

To provide orthogonal confirmation of mosaicism, ultrasensitive digital droplet polymerase chain reaction (ddPCR) assays were designed to validate each putative mosaic MMR variant with evidence from deep sequencing of samples using capture A or B.

Study participant details are provided in Supplementary Table 1. The results from the deep sequencing and ddPCR testing are summarized in Supplementary Table 2. For 3 of 135 participants deep sequenced with capture A, there was evidence of the variant identified in the tumor present in the blood-derived DNA sample, but only 1 of 3 of these variants could be confirmed by ddPCR (Figure 1A). For 2 of 26 participants deep sequenced with capture B, there was evidence of the variant identified in the tumor being present in the normal colon/endometrium DNA, but only 1 of 2 of these variants could be confirmed by ddPCR (Figure 1A). For Patient 151 (EC at 55years and CRC at 57years), the *MSH6* c.1135_1139del p.Arg379* variant present in both the CRC and EC was also detected in DNA sources of different germ layer origin, confirming a soma-wide mosaic *MSH6* variant (Figure 1B, reported previously).^6^

For patient 328 (CRC at 41 years and breast cancer at 54 years), ddPCR analysis confirmed the presence of the *MSH2* c.1413del p.Lys471Asnfs*11 somatic variant in the CRC (67.4% variant allele frequency [VAF]) in the normal adjacent tissue (0.5% VAF) and in the normal nonadjacent tissue 120 mm away from the CRC (0.2% VAF). Normal tissue DNA from other locations in the colon, breast, or blood-derived DNA did not show evidence of the *MSH2* variant (Figure 1B and C), making this the first report of localized MMR gene mosaicism.

Six MMR mosaic cases have now been reported (summarized in Walker et al^6^), including the 2 cases presented here. All 6 MMR mosaic variants were deletions; 4 were a 1-base pair deletion of A purine nucleotide, 1 was a 2-base pair deletion of AT dinucleotides, and 1 was a 5-base pair deletion of AGAGA, with the latter 2 occurring within short tandem repeats (Figure 1D). These specific mutation types suggest a mechanism involving a strand-slippage error during DNA replication or depurination.^8^ Phenotypically, these 6 mosaic cases were predominantly women (5 of 6), ranged in age at first cancer diagnosis from 31 to 79 years (mean ± standard deviation, 51.4 ± 13.0 years), and had ≥2 primary cancers, although this cancer multiplicity is potentially biased due to the ascertainment of cases for testing.

In conclusion, the prevalence of soma-wide MMR mosaicism was <1% (1 of 135), and we provided the first report of localized MMR mosaicism (prevalence <4% [1 of 26]). For the participants found not to have mosaicism, our findings support the likelihood that their MMR-deficient cancer is not caused by Lynch syndrome. Determining localized vs soma-wide mosaicism has important clinical implications; namely, (1) the risk of a second primary CRC for localized MMR mosaics could be mitigated by surgical removal of the affected part of the colon; (2) soma-wide MMR mosaics would have an increased risk of Lynch syndrome-related extracolonic cancers, whereas localized MMR mosaics would not; and (3) the MMR mosaic pathogenic variant would not be present in the primordial germ cells and, therefore, not heritable in localized MMR mosaic cases, whereas this would need to be established for the soma-wide cases.

The low prevalence and multiple tests needed to identify postzygotic MMR gene mosaicism suggests that a targeted approach is needed to select cases rather than systematic screening. MMR-deficient cancers with double or single somatic MMR mutations, where 1 of the mutations involves a purine nucleotide deletion, could be prioritized for deep next-generation sequencing of DNA from normal nonadjacent tissue in preference to blood-derived DNA. Validation of the deep sequencing findings using ddPCR in DNA from different germ layers would then be essential to differentiate localized vs soma-wide mosaicism. Multiple MMR-deficient cancers demonstrating the same pattern of MMR protein loss or with the same somatic MMR mutation would be an important “red flag” for postzygotic mosaicism testing, whereas age at cancer diagnosis appears to be less of an indicator in the few cases identified to date. The identification of additional MMR mosaic cases will help to refine the optimal triaging approach.

## Supplementary Material

1

## Figures and Tables

**Figure 1.**
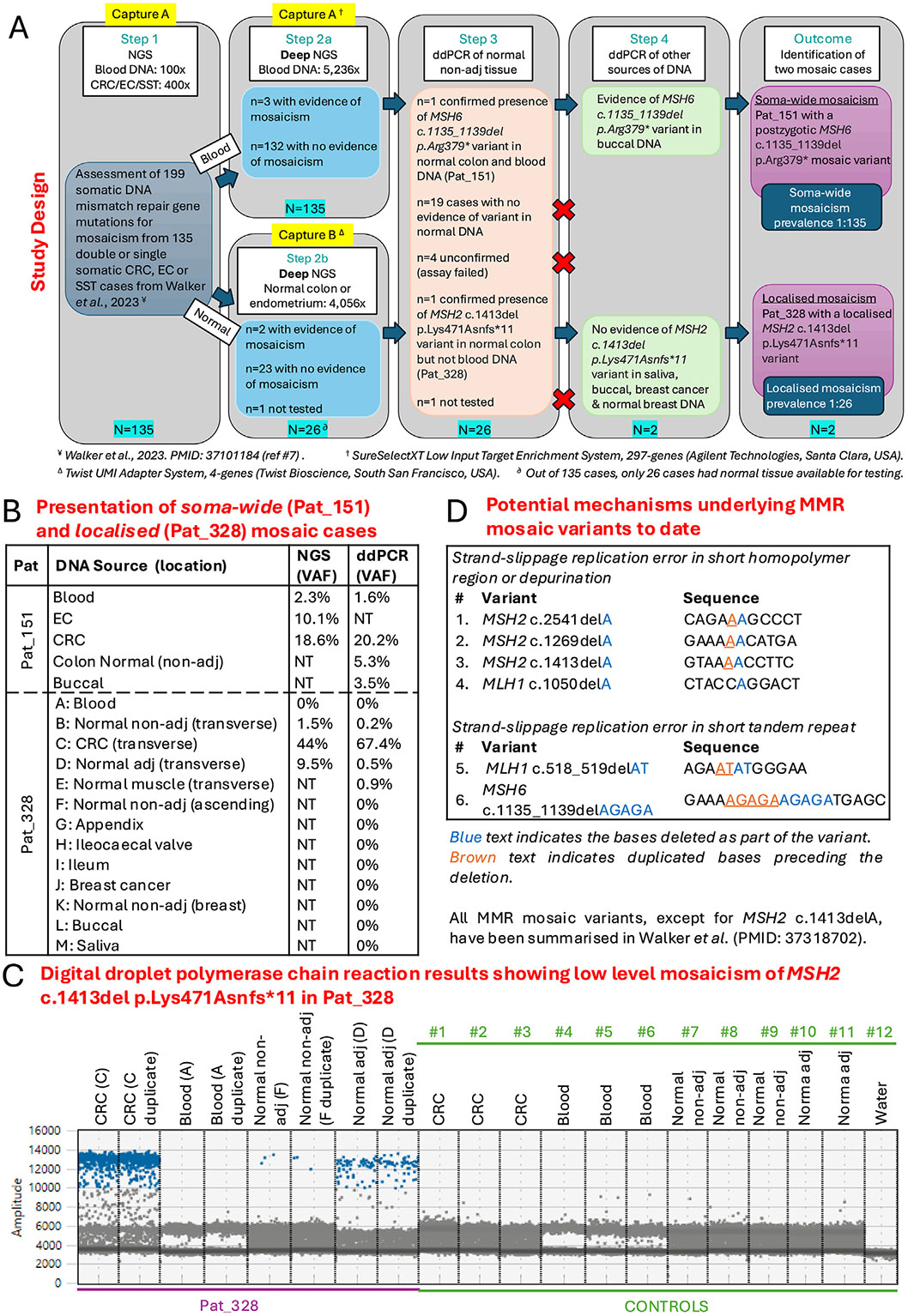
(*A*) Overview of study design and summary of results of MMR mosaicism testing of participants from the ANGELS, CCFR, and the MTS studies. NGS, next-generation sequencing. (*B*) Deep next-generation sequencing and ddPCR results from the *MSH6* c.1135_1139del p.Arg379* soma-wide variant in patient (Pat) 151 and the *MSH2* c.1413del p.Lys471Asnfs*11 mosaic variant from patient 328 shown to be evident in tissue from the transverse colon (samples B–E) but absent from other tissues tested (samples A and F–M) indicating localized mosaicism. (*C*) Results from ddPCR for CRC, normal tissue, and blood DNA samples (run in duplicate) from patient 328 and from 11 nonmosaic individuals included as controls, which were selected to present in the Figure from a larger group of 21 controls, demonstrating evidence of the *MSH2* c.1413del p.Lys471Asnfs*11 mosaic variant in the samples from patient 328 but no evidence of the variant in the control samples. (*D*) Table of the 6 reported MMR mosaic cases, including the 2 from this study, showing the predominance of deletion variants involving an A purine nucleotide and the DNA sequence context 5' and 3' of the affected base. The proposed mechanisms underlying the deletion mutations include strand-slippage replication error or depurination.

## Data Availability

The data generated from the ANGELS and MTS studies are available from Romy Walker upon reasonable request. CCFR data reported in this paper are available via an Application for Collaboration (https://coloncfr.org/for-researchers/collaborate-with-the-ccfr/, last accessed date: September 17, 2024).

## Abbreviations used in this paper:

ANGELS: Applying Novel Genomic approaches to Early-onset and suspected Lynch Syndrome colorectal and endometrial cancers
CCFR: Colon Cancer Family Registry
CRC: colorectal cancer
ddPCR: droplet digital polymerase chain reaction
EC: endometrial cancer
MMR: DNA mismatch repair
MTS: Muir-Torre syndrome
SST: sebaceous skin tumor
VAF: variant allele frequency
